# Amyloidosis of lacrimal gland: Authors’ reply

**DOI:** 10.4103/0301-4738.67059

**Published:** 2010

**Authors:** Sowmya Raveendra Murthy, Kalpana Babu, Anitha Mahadevan, Venkatesh C Prabhakaran

**Affiliations:** 1 Department of Orbit and Oculoplastic Surgery, Prabha Eye Clinic and Vittala International Institute of Ophthalmology, Bangalore, India; 2 Department of Ophthalmic Pathology, Prabha Eye Clinic, Bangalore, India; 3 Department of Neuropathology, NIMHANS, Bangalore, India

Dear Editor,

We thank Kumar *et al*[1] for their interest in our article[2] and the comments.

We do agree that cases reported from the series by Leibovitch *et al*.[3] and Taban *et al*.[4] were not included, and we also agree that the bilateral case reported by Cheng *et al*.[5] was not included in the discussion. However, the case described by Knowles *et al*.[6] was orbital amyloidosis, than mere lacrimal gland involvement.

We would like to clarify that the axial proptosis was 2 mm and 2 mm was the inferior displacement.

We do agree that debulking is essential. However, this patient only had an incisional biopsy for diagnosis and opted to be under follow-up.
